# Characterization of high psychiatric emergency department users - Unidade Local de Saúde Viseu Dão-Lafões, Portugal, from 2022 to 2024

**DOI:** 10.1192/j.eurpsy.2026.10941

**Published:** 2026-07-31

**Authors:** R. P. L. Andrade, I. Santos, J. Brás, B. Melo, H. Afonso

**Affiliations:** Psiquiatria, ULS Viseu Dão-Lafões, Viseu, Portugal

## Abstract

**Introduction:**

Frequent emergency department users are high-risk and high-resource-utilizing patients. In psychiatry, reasons for frequent use of psychiatric emergency departments (PED) are complex, numerous and unpredictable.

**Objectives:**

This study aimed to identify profiles of high PED users (4+ visits/year) based on their patterns of PED use and clinical characteristics and to evaluate demographic factors associated with these patients.

**Methods:**

This was a retrospective cohort study of all patients presenting to Unidade Local de Saúde Viseu Dão-Lafões PED, from January 2022 to December 2024.

**Results:**

In 2022, 34 patients were identified as high PED users, accounting for a total of 194 episodes. 58.8% were female, and 50% of them live in the city where the hospital is located. Regarding diagnosis, 47.1% had comorbidity with Personality Disorders (PD), 23.5% had comorbidity with Disorders of Intellectual Development (DID), and 23.5% had comorbidity with Psychiatric Disorders with psychotic symptoms. Of the 194 episodes, 22 resulted in hospitalization.

In 2023, 23 patients were high PED users, accounting for a total of 142 episodes. 52.2% were female and 60.9% of them lived in the city where the hospital is located. Regarding diagnosis, 39.1% of users had comorbidity with PD, 26.1% had comorbidity with DID, and 26.1% had comorbidity with Psychiatric Disorders with Psychotic Symptoms. Of the 142 episodes, 20 resulted in hospitalization.

In 2024, 30 patients were identified high PED users, accounting for a total of 194 episodes. 43.3% were female and 56.7% lived in the city where the hospital is located. Regarding diagnosis, 30% of users had comorbidity with PD, 23.3% had comorbidity with DID, and 33.3% had comorbidity with Psychiatric Disorders with Psychotic Symptoms. Of these 194, 20 resulted in hospitalization.

**Conclusions:**

With this work, it was possible to verify that in 2022 and 2023, female users resorted more to the PED, as high users, a context that was reversed in 2024. Also in 2024, there was a greater preponderance of users with psychiatric disorders with psychotic symptoms, relative to PD and DID, for example, compared to 2022 and 2023.

Nevertheless, the fact that in that year, the 194 emergency episodes involving high PED users resulted in only 20 hospitalizations suggests that most of these episodes did not actually correspond to genuine psychiatric emergencies. Finally, easy access to the hospital, such as proximity to it, may increase the number of emergency episodes.

Therefore, it will be important to discuss strategies to be implemented to reduce the number of non-emergency visits to the PED.

**Disclosure of Interest:**

None Declared

